# Best Practices for Progressive Return to Activity after Concussion: Lessons Learned from a Prospective Study of U.S. Military Service Members

**DOI:** 10.1089/neur.2020.0023

**Published:** 2020-10-29

**Authors:** Mark L. Ettenhofer, Rosemay A. Remigio-Baker, Jason M. Bailie, Wesley R. Cole, Emma Gregory

**Affiliations:** ^1^ Defense and Veterans Brain Injury Center, Silver Spring, Maryland, USA.; ^2^ Naval Medical Center San Diego, San Diego, California, USA.; ^3^ General Dynamics Information Technology, Fairfax, Virginia, USA.; ^4^ University of California, San Diego, La Jolla, California, USA.; ^5^ Naval Hospital Camp Pendleton, Camp Pendleton, California, USA.; ^6^ Henry M. Jackson Foundation, Bethesda, Maryland, USA.; ^7^ Womack Army Medical Center, Fort Bragg, North Carolina, USA.

**Keywords:** concussion, military, primary care, return to activity, traumatic brain injury

## Abstract

Primary care providers can play a crucial role in the clinical management of concussion. However, many providers lack up-to-date information about best practices for rest and return to activity after these injuries. Most research on this topic has been conducted in athletes, and so less is known about how to assist patients with returning to activity in other settings and populations. This article provides a review of best practices for management of progressive return to activity after concussion, with an emphasis on “lessons learned” from the Defense and Veterans Brain Injury Center (DVBIC) Progressive Return to Activity (PRA) study, a multi-site longitudinal research project conducted to evaluate concussion management practices and the effectiveness of provider training on DVBIC clinical recommendations (CRs). Provider clinical practices and patient outcomes were examined at three U.S. military treatment facilities before and after providers completed a standardized training on DVBIC PRA CRs. In summary, research findings provide additional support that concussion recovery can be influenced by patients' activity levels after injury. Patients with concussion may experience poorer outcomes if they return to pre-injury levels of activity too rapidly, but they may also be at risk for prolonged symptoms if they fail to increase activity levels over time after an initial period of rest. Additionally, training primary care providers in return to activity guidelines can result in more effective patient education and better clinical outcomes. This knowledge can be used to inform best practices for progressive return to activity in both civilian and military settings.

## Introduction

Concussion is a very common condition, with more than 600 such injuries per year for every 100,000 persons globally.^1^ Certain groups, such as athletes^2,3^ and military service members (SMs)^4–6^ are at particular risk for concussion. Although most concussions resolve within the first few weeks to months following injury, a subset of these patients require more extensive clinical management and experience significantly reduced productivity.^7^ With proper training, primary care providers can play a crucial role in preparing patients with concussion for a successful recovery. However, many clinicians have difficulty remaining up to date about concussion recovery and empirically supported approaches to treatment. As we describe below, evidence suggests that targeted training in best practices for concussion management can provide a substantial boost to patients' recovery.

Studies conducted in sport settings have provided valuable opportunities to identify concussions rapidly, to closely track patients before and after injury, and to monitor post-injury activities such as rest, training/practice, and return to play. This research has heavily influenced current clinical practice guidelines for concussion management. The U.S. Military Health System provides an additional point of reference to inform best practices for concussion management among SMs, and, likely, for other concussed individuals. The U.S. military population includes an estimated 2.2 million active duty and reservist SMs, and bears many similarities to populations of athletes, but also differs in some important ways. Like athletes, many SMs' jobs are physically demanding, but job duties can vary widely across combat and support roles—including many roles that mirror common occupations within the civilian workforce. SMs frequently endure high-stress operations for extended periods of time, and concussed SMs are more likely to have pre-existing or co-occurring conditions such as multi-system injuries, post-traumatic stress, depression, and anxiety.^6,8^ Additionally, although many SMs are active in athletics, the majority of SMs' concussions are sustained via motor vehicle accidents, training accidents, and workplace accidents/falls.^9^ Each of these factors may influence individual medical needs and the trajectory of recovery after injury.

The unique characteristics of military SMs have prompted concussion research targeted to the needs of this population. In 2015, following publication by the Defense and Veterans Brain Injury Center (DVBIC) of “Progressive Return to Activity Following Acute Concussion/Mild Traumatic Brain Injury: Guidance for the Primary Care Manager in Deployed and Non-deployed Settings,”^10^ DVBIC launched the Progressive Return to Activity (PRA) study.^11^ This multi-site longitudinal research project collected information about primary care providers' clinical practices and concussion patients' outcomes at three major U.S. military treatment facilities serving U.S. Army, Navy, and Marine Corps populations. The principal goals of the project were to improve understanding of concussion care and recovery for active duty SMs, and to identify methods to enhance patient outcomes by increasing adherence to clinical recommendations (CRs) for PRA after injury. The results of this research have informed clinical recommendations for concussion management within the U.S. military, and may also provide valuable information for improving care across other populations sustaining concussions. In the following sections, we review important information relevant to return to activity and recovery after concussion, with an emphasis on lessons learned from the DVBIC PRA study.

### Changes in neurobehavioral symptoms and activity levels across the course of concussion recovery

In addition to the neurological signs that form the basis of concussion diagnosis (loss of consciousness, alteration of consciousness, and/or post-traumatic amnesia), acute concussion can result in a wide range of neurobehavioral symptoms, such as headache, dizziness, fatigue, irritability, and cognitive difficulties. In most individuals, these symptoms reduce substantially over the first few days and weeks after injury. However, the time frame for symptom recovery can be highly variable. Although research in sport settings suggests that most athletes recover from concussion within 7 days,^12–14^ adults admitted to the emergency room (ER) for concussion may continue to report elevated symptoms 6 months^15^ or 1 year after injury.^16,17^

**FIG. 1.**
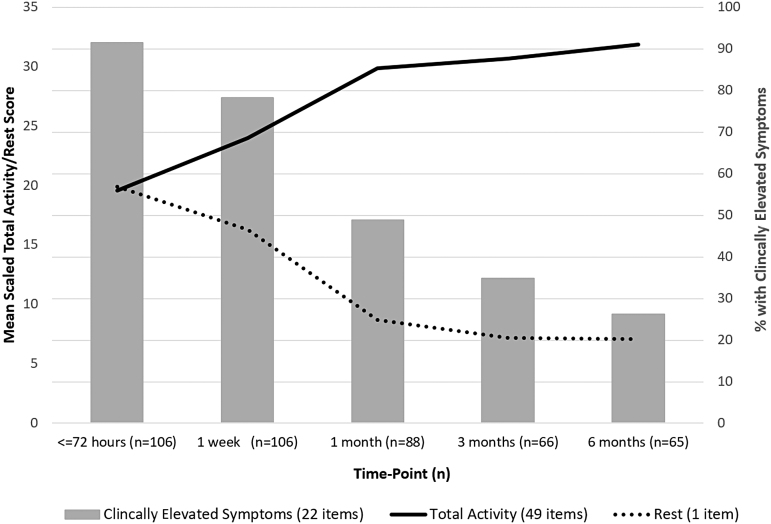
Activity levels and rates of clinically elevated post-concussive symptoms over time. Clinically elevated symptoms are defined as a Neurobehavioral Symptom Inventory score >75th percentile based on normative data adjusted for self-reported deployment history.^18^

Data from the DVBIC PRA study illustrate the trajectory of recovery among active duty SMs sustaining concussion in non-deployed environments (see Fig. 1). A large majority (91.5%) of concussed SMs in this study displayed clinically elevated levels of post-concussive symptoms during their initial visit (<72 h post-injury). A clinically elevated level of post-concussive symptoms is defined as a Neurobehavioral Symptom Inventory score >75th percentile based on normative data adjusted for self-reported deployment history. Almost half (48.9%) were still clinically symptomatic 1 month later, but this dropped to 34.5% by 3 months post-injury. By 6 months post-injury, the proportion of individuals with elevated symptoms declined to 28.8%, which was in line with what would be expected in an uninjured military population with one or more combat deployments.^18^ Overall, these results suggest that concussion recovery among active duty SMs may be less favorable than outcomes demonstrated in previous studies of athletes, but similar to findings from civilian ER samples.^15^

Post-concussive symptoms can cause distress, reduce quality of life, and interfere with participation in important life activities. However, relatively few studies have examined the trajectory of return to activity after concussion outside of athletic settings.^19–24^ Details about appropriate timeline and scope for returning to activity are particularly salient for SMs, whose duties may include a wide range of stressful and potentially dangerous activities.^25^

**FIG. 2.**
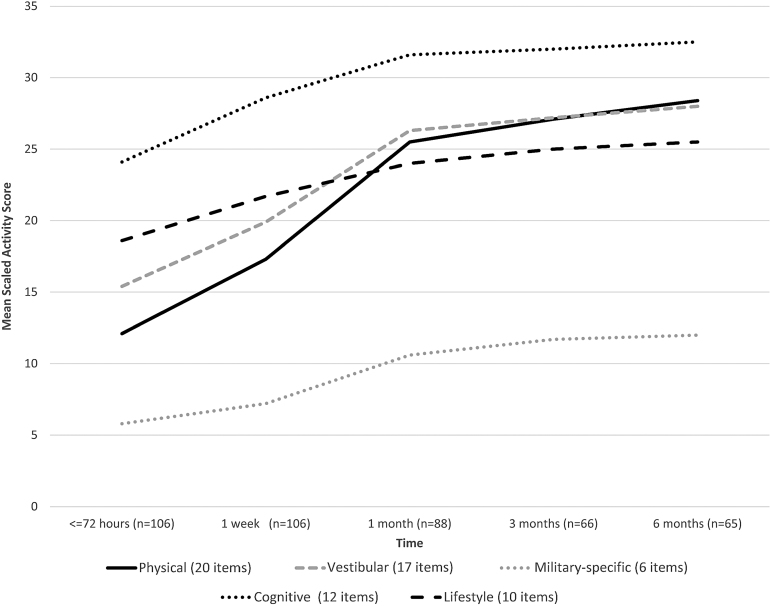
Changes in activity levels over time after concussion, by type of activity.

As shown in Figure 1, concussed SMs in the DVBIC PRA study engaged in relatively low levels of activity (and high levels of rest) immediately after injury, followed by gradual increases in activity over the following 3 to 6 months, with an average increase in total activity of 63% over the full study time frame.^26,27^ Physical, vestibular/balance, and military-specific activities increased most rapidly, whereas cognitive and lifestyle activities (i.e., reading, socializing, watching television) increased more slowly (Fig. 2). The tendency for activity levels to increase as post-concussive symptoms decrease reflects a complex interplay of factors with important implications for management of patients with concussion. First, individuals with concussion may avoid activities that they believe might exacerbate their symptoms, increasing activity only as their symptoms permit. Additionally, a growing body of evidence suggests that the type, timing, and level of a patient's activities can have a significant impact on the speed of concussion recovery.^19–24,28,29^

### Level of activity influences recovery from post-concussive symptoms

Actively managing concussion patients' return to activity may be one of the most powerful ways to facilitate a speedy and complete resolution of symptoms. Some studies have found that returning to activity prematurely (i.e., prior to resolution of symptoms) is associated with worse symptoms or longer recovery time.^19–21,23,24,29^ Accordingly, clinical practice guidelines suggest that acutely concussed individuals should complete an initial period of rest and avoid physically or mentally strenuous activities that could aggravate their symptoms or increase risk of re-injury.^10,30,31^ However, the details of these guidelines have been based largely on expert opinion/consensus rather than objective data, and some controversy remains regarding best practices for return to activity within specific populations and settings.

Results from the DVBIC PRA study provide additional support for the effectiveness of activity restrictions during the initial phase of concussion recovery. Within this sample of concussed SMs, activity levels reported at <72 h post-injury were highly variable, ranging from almost total rest to high levels of physical, vestibular/balance, cognitive, lifestyle, and military-specific activity.^26^ Those who returned to activity prematurely (i.e., those reporting high levels of symptoms *and* high levels of activity in the first few days after injury) had higher levels of symptoms 1, 3, and 6 months later. Interestingly, the negative effects of excessive activity were most prominent for vestibular symptoms, such as dizziness or loss of balance.^26^ Results also suggested that patients who did not believe in the importance of rest after concussion experienced slower resolution of symptoms.^32^

However, providers who over-emphasize rest—and those who fail to track their concussed patients beyond the initial phase of injury—may also overlook critical opportunities to optimize recovery, as excessive rest during post-acute stages of concussion has been shown to prolong symptoms.^29,30,33^ Additionally, an emerging literature suggests that guided physical activity can be an effective intervention in the post-acute and chronic phases of recovery.^34,35^ Results from the DVBIC PRA study provided additional support for these findings, as SMs who engaged in higher levels of physical or vestibular activity during the sub-acute stage (1 month post-injury) reported lower levels of symptoms in the chronic stage (3 months post-injury).^36^ This apparent reversal in the effects of activity over time highlights the importance of matching concussion patients' activity levels to their stage of recovery, with a graded increase in activity level as symptoms subside.^10,33,37–42^ Although implementing PRA may require additional monitoring and management throughout the acute and sub-acute stages, these extra efforts are likely to translate into faster recovery and lower rates of chronic issues—particularly for patients who were highly symptomatic during the acute stage.

Results from the DVBIC PRA study also provided valuable clues about the types of activity that have the greatest impact on concussion recovery. Most notably, DVBIC PRA study participants' involvement in physical or vestibular activities during early/acute concussion, such as lifting weights and performing agility drills, were linked to delayed symptom resolution.^26^ Fortunately, evidence supports the value of vestibular rehabilitation for those experiencing these symptoms,^43–45^ and within the DVBIC PRA study, engagement in vestibular activities later (in the sub-acute phase) was associated with faster recovery.^36^ Although only a small subset of patients with concussion are likely to receive targeted rehabilitation services, the guidance and structure of a graded return to activity provided by front-line medical personnel may be valuable. This includes discouraging premature return to activity during the acute stage to prevent acute symptoms from becoming chronic, and in treating patients whose symptoms have begun to linger by encouraging resumption of appropriate activities during post-acute phases of recovery.

### Enhancing patients' adherence to return to activity guidance

As with any treatment, effectiveness of a return to activity plan depends on patients' adherence to the guidance provided. Although the likelihood that a patient will comply with recommendations for activity (i.e., about 35% for exercise plans for managing chronic illnesses^46^) tends to be even lower than the likelihood that they will take a medication as directed (about 50%^46,47^), evidence suggests that effective patient education can substantially increase adherence to medical guidance. This is especially relevant to concussion treatment, as patients' knowledge, beliefs, and expectations about concussion can have a major impact on clinical outcomes after injury.^32,48,49^

For example, groups of patients that *expect* more severe, longer-lasting symptoms after concussion are more likely to actually experience these negative outcomes.^50^ Likewise, educational and behavioral interventions aimed at correcting misconceptions and providing realistic expectations for recovery have been found to be effective within both acute and chronic concussion populations.^51–54^ Results from the DVBIC PRA study show how these previous findings may apply to implementation of PRA guidelines within a primary care setting: patients who received education from their providers during the acute stage of injury were not only more likely to believe that early rest is important to concussion recovery, but they were also more likely to increase their levels of activity appropriately over time, and they experienced a more rapid reduction in symptoms than patients who did not receive education.^27,32^

### Training providers on progressive return to activity guidelines

As discussed, information about stage of concussion recovery can be used to guide patients through the process of PRA and may hasten their overall recovery toward pre-injury levels of functioning. However, many providers are responsible for providing care for a wide range of different conditions, and so they may lack up-to-date knowledge of best practices for management of concussion. In a second phase of the DVBIC PRA study, we sought to evaluate the effects of directly training primary care providers on the DVBIC PRA CR.^11,27,32^

Although the DVBIC PRA CR was publicly available online prior to training, providers indicated relatively low rates of exposure to this information before enrollment in the study. Concussion management clinical practices (including the likelihood of providing patient education) varied among primary care managers, both within and between the three military treatment facilities investigated. This highlighted the potential value of a standardized approach, and informed the development of a standardized, targeted (2 h) in-person training on the DVBIC PRA CR to be utilized across military providers and military treatment facilities. The interactive training included a detailed overview of the content of the PRA CR, as well as small-group case study activities, a workbook, and written reference materials. Provider clinical practices and patient outcomes were examined before and after providers completed the training.^11^

**FIG. 3.**
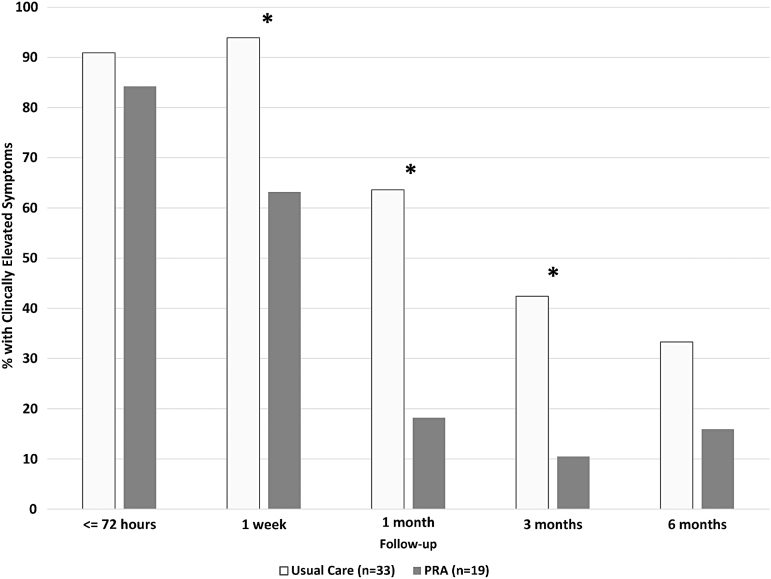
Effects of provider training (progressive return to activity [PRA]-trained provider vs. usual care) on rates of clinically elevated symptoms over time. Clinically elevated symptoms are defined as a Neurobehavioral Symptom Inventory score >75th percentile based on normative data adjusted for self-reported deployment history.^18^ *Significant difference at a *p*-value <0.05. Adapted from a study by Bailie and colleagues.^27^

Results suggested that this training had a significant positive impact on clinical practices as well as on patients' recovery from concussion.^27^ Interview data and informal feedback indicated that the training was well received and impacted concussion clinical practices as intended. Quantitative results were even more compelling; compared with patients who received concussion treatment before providers received the PRA training, patients who were treated after provider training were 59% more likely to receive education from their providers, and they had lower levels of physical and vestibular activity during the first week of concussion recovery.^27^ Importantly, these differences translated into expedited recovery.^27^ Despite comparable levels of symptoms immediately after injury, symptomatic patients receiving care from providers who had received PRA CR training exhibited better symptom recovery at 1 week, 1 month, and 3 months after injury, as compared with similar patients who received care from providers who had not yet completed PRA CR training^27^ (see Fig. 3). Overall, these results provide evidence that primary care providers can play a powerful role in concussion recovery, and that training these providers in best practices for return to activity can help to maximize their positive impact on patient outcomes.

### Additional factors to consider in planning return to activity after concussion

The DVBIC PRA study focused on providers' clinical practices, patients' activity levels, and the influence of education on these outcomes. However, there are several additional factors to consider when treating concussion. A wide range of prior or concurrent conditions can increase risk of poor outcomes after concussion, including comorbid physical conditions,^55^ history of migraine,^55^ pre-injury psychiatric conditions,^55,56^ and history of learning disabilities/attention deficit hyperactivity disorder(ADHD).^57,58^ Additionally, history of prior concussions has been associated with slower recovery from a new concussion.^59–62^ Accordingly, the DVBIC PRA CR indicates that patients experiencing more than one concussion within a year should be treated more conservatively, with longer delays between progressive activity stages and/or more rapid referral for specialty services.^10^ Continued research may yield information about additional factors that can impact the trajectory of recovery after concussion. Clinical outcomes may benefit from an increase in the level of provider monitoring and management for patients with conditions associated with elevated risk for persistent symptoms.

## Conclusion

As an “invisible injury,” concussion can be a confusing and worrisome condition for patients and providers alike. Results from the DVBIC PRA study provide evidence that primary care providers can play a crucial role in concussion management and patient education. Further, although some groups, such as athletes, typically recover quickly, other groups, such as civilians and SMs, may take longer for their symptoms to resolve. Providers should consider a wide range of factors that may modify speed of recovery, such as patients' activity levels, their beliefs/expectations about concussion, and their prior medical history. After an initial period of rest, patients' level of activity should progressively increase in tandem with remission of symptoms. Patients with concussion may have poorer outcomes if they return to pre-injury levels of activity too rapidly, but they are also at risk for prolonged symptoms if they fail to increase activity levels over time (i.e., by 1 month post-injury). Providers who are directly trained in return-to-activity guidelines are more likely to provide effective patient education, ultimately helping their patients to recover more rapidly.

## Abbreviations Used

CR: clinical recommendation
DVBIC: Defense and Veterans Brain Injury Center
ER: emergency room
PRA: progressive return to activity
SM: service member
